# MicroRNA-411 inhibits malignant biological behaviours of colorectal cancer cells by directly targeting PIK3R3

**DOI:** 10.3892/or.2022.8283

**Published:** 2022-02-11

**Authors:** Jian Zhao, Jian Xu, Rui Zhang

Oncol Rep 39: 633-642, 2018; DOI: 10.3892/or.2017.6135

Following the publication of the above article, the authors have requested that it be retracted on account of supplementary experiments needing to be performed to verify some of the results. Furthermore, following a further investigation in the Editorial Office, it came to light that some of the cell apoptotic data shown in Figs. 2, 5 and 6 were strikingly similar to those that had been submitted for publication prior to the receipt of present article. Therefore, this article has been retracted from the Journal; all the authors agree to this retraction. The Editor and the authors would like to apologize for any inconvenience caused.

